# A Historic Report of Zika in Mozambique: Implications for Assessing Current Risk

**DOI:** 10.1371/journal.pntd.0005052

**Published:** 2016-12-08

**Authors:** Eduardo Samo Gudo, Kerstin I. Falk, Sadia Ali, Argentina Felisbela Muianga, Vanessa Monteiro, Julie Cliff

**Affiliations:** 1 Virus Isolation Laboratory, National Institute of Health, Ministry of Health, Maputo, Mozambique; 2 Department of Microbiology, The Public Health Agency of Sweden, Solna, Sweden; 3 Department of Microbiology, Tumor and Cell Biology, Karolinska Institute, Solna, Sweden; 4 Community Health Department, Faculty of Medicine, Eduardo Mondlane University, Maputo, Mozambique; Oswaldo Cruz Foundation, BRAZIL

## Background

Zika virus (ZIKV) is an arthropod-borne flavivirus transmitted to humans by the bite of the *Aedes* mosquito. The virus was first isolated in 1947 from a rhesus monkey in the Zika forest in Uganda [1].

For more than half a century, the virus was confined to Africa and Asia, and only 14 human cases of Zika had been documented [2]. This changed in 2007 when an outbreak was reported on Yap Island in Micronesia [3, 4], followed by a large epidemic in French Polynesia in 2013 [5, 6]. More recently, ZIKV has spread explosively in South and Central America and the Caribbean [2, 7]. Reports of associated microcephaly cases and other neurologic disorders in Brazil and a similar cluster in French Polynesia in 2014 led WHO to declare a Public Health Emergency of International Concern (PHEIC) on February 1st, 2016 [8].

To prevent globalization of ZIKV, WHO developed a Zika strategic response framework. They recommended that unaffected countries rapidly assess their risk of being hit by ZIKV [9] in order to develop preparedness plans. The fear of ZIKV is attributed to the fact that not only has *Aedes*, the main vector of ZIKV, spread exponentially over the world in the last decades but also because of the large number of susceptible populations worldwide and the absence of an effective vaccine [9, 10].

## Zika in Sub-Saharan Africa

The sub-Saharan Africa region where the first cases of Zika in humans were initially reported is the least prepared but is at most risk. Since its discovery in 1947, sporadic cases of Zika have been reported in several countries in sub-Saharan Africa [2]. So far in sub-Saharan Africa, only Cape Verde has reported a recent outbreak, with more than 5,000 suspected cases [11].

However, for several decades the presence of ZIKV was only sporadically studied in Southern Africa, since Zika was considered a benign disease with a low public health importance. In addition, few countries have performed mapping of the distribution of *Aedes*, which represents an important gap in assessing the risk of spread of the virus to these countries. Most of the reported human cases of Zika are situated in Central, East, and West Africa [1, 12–19].

With regard to the Southern Africa region, serosurveys have been conducted in Angola, Tanzania, Zambia, and Mozambique, which have found positive results for ZIKV; no other data are available [2]. However, most of these studies are quite old and most were conducted between the 1960s and 1990s [2].

## Past History of Zika in Mozambique and Implication for Assessing Current Risk

Although Kokernot et al. found neutralizing antibodies against ZIKV in Mozambique in 1957 [20], the country has repeatedly been excluded from the list of countries with a past history of ZIKV. The current Centers for Disease Control and Prevention map, the recent World Health Organization Risk Assessment map on ZIKV in the Africa region, and most of the recent literature mapping countries with a current and past history of ZIKV have consistently excluded Mozambique [10, 21, 22] from the list of countries with past serological evidence of ZIKV. This might result in errors in the calculation and interpretation of the risk of Zika in Mozambique as well as in the region. In this regard, in this manuscript we revisit findings of the study conducted by Kokernot et al. in an attempt to discuss the current risk of Zika in the country. The survey, conducted in 1957, was published in Portuguese in 1960 [20].

This study was part of a larger study on arboviruses, in which blood samples were screened for antibodies against 13 arboviruses, including ZIKV, chikungunya, Rift Valley fever, Sindbis, Middleburg, and Wesselsbron. Samples were collected in 29 localities situated widely apart from each other throughout the country between July and August 1957. In each locality, they selected an average of 30 local residents who had been born in the area with no history of travel outside in their lifetime.

The samples were analyzed in South Africa, using confirmatory neutralization testing (NT). NT was performed using an in vivo system. For this purpose, previously titrated virus strains for each arbovirus being tested were incubated with each participant’s serum and inoculated into Swiss mice to assess the neutralization profile of each serum against each virus strain. For ZIKV, the prototype ZIKV strain was used [18], and both adult and newborn mice were used for inoculation. An amount of 0.03 mL of the preparation virus and serum was inoculated intracerebrally, as previously described [18], and each mouse was observed daily between 10–17 days to assess the viral effect.

The authors found neutralizing antibodies to all of the viruses and concluded that the whole length of Mozambique was a “tropical corridor” of arbovirus activity. The viruses with highest prevalence rates of neutralizing antibodies were chikungunya (21.0%), Wesselsbron (15.9%), Bunyamwera (24.1%), Pongola (23.2%) and Bwamba fever (24.7%).

**Fig 1:**
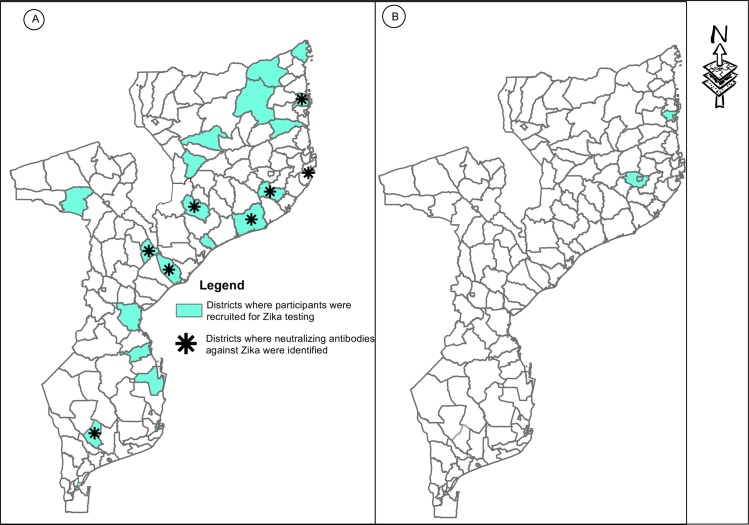
Geographical distribution of districts where study was conducted and where neutralizing antibodies against Zika were found during the Kokernot et al. study in 1957 (panel A) and where recent outbreaks of Dengue were reported (panel B). Credit: Américo Feriano José.

ZIKV was tested for in a more limited number of samples from 22 localities (see Fig 1). Of a total of 249 samples (152 adults, 107 children) tested, the overall prevalence was 4.0% (10/249). Serological findings were analyzed by age, geographical distribution, and altitude. The highest prevalence rate—9.8% (6/61)—was found in adults living in areas below 200 m north of the Zambezi river. The ZIKV seroprevalence rate was lower in children: 1.0% (2/107) compared to 5.6% (8/152) in adults. Neutralizing antibodies against ZIKV were found in nine localities (see Fig 1). For adults, they were found in seven localities—namely, Guijá (1/1), Sena (1/13), Mualama (2/11), Nametil (1/9), Lumbo (1/15), and Quissanga (2/13). For children, antibodies were found in two localities—namely, Marromeu (1/5) and Belua (1/1). The prevalence rate was slightly higher south of the Zambezi river compared to north of the river (4.2%, 3/71 south versus 3.9%, 7/178 north). This study also showed that the prevalence of ZIKV was higher in places situated at an altitude between 0–200 m (5.2%, 9/173) compared with those situated between 200–1000m (1.3%, 1/73).

These findings should be interpreted with caution, as cross-reaction between flaviviruses has been reported in NT assays. To help clarify this issue, we revisited carefully the original manuscript of ZIKV isolation published by Dick et al. in 1952 [18] and noted that dengue immune serum had no relevant neutralizing effect on ZIKV using an in vivo NT assay similar to that used by Kokernot et al. [18].

Our belief is that the manuscript published by Kokernot et al. has been consistently ignored in most of the international literature because although the title of the manuscript is available in English on PubMed, it is not found using keywords such as “arbovirus and Mozambique” or “Zika and Mozambique,” possibly because of deficient indexing of old articles.

Old literature is relevant, particularly at a time when countries are developing their national preparedness plan for Zika and microcephaly. In this regard, the exclusion of Mozambique from the list of countries with past history of serological evidence of ZIKV might hamper national and international efforts to prevent Zika and microcephaly in the country.

While the 1957 study in Mozambique suggests that ZIKV may have circulated in the past, we cannot assume that the virus is still present. No further studies have been conducted in Mozambique, which limits our discussion on whether the virus may have been eliminated during the efforts in the 1970s and 1980s to reduce the malaria burden through massive distribution of bed nets, community education, indoor and outdoor spraying campaigns, and appropriate malaria case management, among others [23, 24]. Whether efforts to reduce the burden of *Anopheles* mosquitoes in Mozambique resulted in any impact in the population of *Aedes* is unknown as, for several decades, most entomological investigation in Mozambique was restricted to *Anopheles*. During these decades, *Aedes* was never considered during vector surveillance activities until recently, when a dengue outbreak hit two provinces in northern Mozambique and *Aedes* was found to be abundant in northern Mozambique [25].

Whether the past evidence of antibodies against ZIKV in Mozambique suggests that the country is vulnerable to the current virus strain circulating in the Americas is not known. The ZIKV has evolved since it was first isolated in Uganda in 1947 [14, 26]. The African lineage is slightly different from those circulating in Asia and the Americas [2, 14]. Whether the African lineages of ZIKV have a lower ability to spread and cause outbreaks or epidemics in sub-Saharan Africa is still a matter of debate.

On the other hand, the initial descriptions of ZIKV in mosquitoes were linked to other species of *Aedes*, such as *Aedes africanus*, *A*. *furcifer*, *A*. *taylori*, and *A*. *luteocephalus* [27]. The occurrence of sporadic human cases of Zika reported in West and Central Africa, and the absence of cases further south, may suggest lower virus competence. Notably, a recent study on *Aedes* competence to assess its ability for transmitting ZIKV found that *Aedes* had a lower to null competence to transmit the virus [28]. Chouin-Carneiro et al. had a recent publication in which they found a lower competence of both *A*. *aegypti* and *A*. *albopticus* from the Americas in transmitting ZIKV [29]. A recent editorial in the *South African Medical Journal* on the risk of Zika in South Africa observed that prediction models using the distribution of *A*. *aegypti* do not place that country at high risk [30].

Kokernot et al.’s serosurvey was accompanied by a survey for culicine mosquitoes [31]. The results, published with a review of other information, showed that *Aedes* was present in many locations in Mozambique. They included a published study conducted in 1959 and 1960 in Lumbo, situated in northern Mozambique, which found *Aedes* [32]. Our recent study conducted in April 2014 in four large cities in Mozambique and aiming to investigate the presence and abundance of the *Aedes* mosquito found that *Aedes* occurred in all four cities. The cities were Pemba, Nampula, and Nacala in northern Mozambique and the capital of Maputo Province in southern Mozambique [33]. The proportions of sampled tires containing *Aedes aegypti* were 72.0% in Pemba, 59.0% in Nampula, and 64.5% in Nacala, compared to 19.3% in Maputo Province. Thus, *Aedes aegypti* was more abundant in the three northern cities compared to the southern [33]. The greater abundance of *Aedes aegypti* together with a higher proportion of *Aedes* in the northern cities suggests that arboviral transmission may be greater in northern Mozambique. To date, outbreaks of dengue have only been reported from northern Mozambique [25, 34]. This suggests that the risk of Zika may be higher in northern compared to southern Mozambique.

Taken together, the historical data on Zika, a recent outbreak of dengue [25], and the recent detection of chikungunya antibodies in febrile patients in southern Mozambique [35] highlight an urgent need to implement vector surveillance and control of *Aedes* mosquitoes as part of arbovirus control in Mozambique. *Aedes* surveillance and control activities have been heavily neglected in Mozambique in recent decades, with entomological interventions entirely focused on malaria. Since vector surveillance and control activities for malaria are well established in Mozambique, we recommend that *Aedes* control activities should be integrated into the existing activities for malaria control in order to maximize the scarce resources. Our findings also suggest that particular attention should be given to provinces situated in northern Mozambique, where arboviral transmission is probably higher.

The recent confirmation of sexual transmission of ZIKV [36–38] poses a serious concern for countries with a high incidence of sexually transmitted diseases such as Mozambique, which occupies the eighth position among the countries with the highest HIV-1/2 prevalence in the world [39, 40]. In addition to spread through an abundance of *Aedes*, the risk of spread through unprotected sexual intercourse should be taken into account.

In regard to Mozambique, no study on vector competence has been conducted so far, which limits our discussion related to vector competence of *A*. *aegypti* circulating in Mozambique. In this regard, although our recent publication showed that *Aedes* mosquitoes were abundant in northern Mozambique [25] and another recent publication also by our group showed that *A*. *albopictus* circulates in southern Mozambique [41], studies of vector competence of *Aedes* mosquitoes are urgently needed to assess the ability of the *Aedes* strains in Mozambique to transmit the current virus strain circulating in the Americas. Moreover, genetic variations of *Aedes* mosquitoes have been shown to lead to variations in the ability of *Aedes* to transmit arbovirus [42, 43]. The literature shows that *Aedes* is highly prevalent in most sub-Saharan African countries, including those that share borders with Mozambique [44–48].

In conclusion, this manuscript combines historic data with recent findings to suggest that arboviral activity may be intense in Mozambique, particularly in the northern part of the country where *Aedes* is abundant and past and recent outbreaks of dengue have been reported. The historic findings reported by Kokernot et al. provide sufficient evidence to include Mozambique in the list of countries at risk of Zika and in need of immediate interventions to generate the missing evidence needed for the design of effective prevention strategies. In view of these findings and taking into consideration the scarcity of funds for arboviral related interventions, we propose (i) incorporation of entomological surveillance for *Aedes* within the framework of existing entomological surveillance for malaria vectors, (ii) incorporation of surveillance for arboviruses within the existing surveillance system for endemic diseases, and (iii) integration of *Aedes* control interventions within the existing framework for controlling the malaria vector. Integration of interventions for surveillance and control of malaria and arboviruses should take into consideration the specificities and difference of the ecology of *Anopheles* and *Aedes* mosquitoes.
